# ACCEPTANCE AND COMMITMENT THERAPY INTERVENTION FOR PARENT CAREGIVERS: A STRENGTH-BASED APPROACH

**DOI:** 10.1093/geroni/igad104.1230

**Published:** 2023-12-21

**Authors:** Miranda Frederick, Jeong Eun Lee, Amie Zarling

**Affiliations:** Iowa State University, Ames, Iowa, United States; Iowa State University, Ames, Iowa, United States; Iowa State University, Ames, Iowa, United States

## Abstract

ACT has emerged as a behavioral change approach that has been applied to address a wide range of challenges. Empirical research has shown that ACT can deliver positive long-term effects across various populations, including caregivers. Using six core principles of ACT, we have revised an acceptance and commitment therapy program (ACT) for custodial grandparents and their grandchildren. Our intervention program emphasized the importance of acceptance and promoting resilience among participants. This approach is particularly valuable for this population as the focus of the program is to strengthen their competencies, life skills and emotional resilience. The program consists of a web based ACT program with online coaching meetings across four sessions delivered for each age group. This program is unique in the sense that it utilizes both individual and group session techniques to facilitate the learning process. The main purpose of the program is to promote effective coping strategies, to reduce parenting stress among grandparents and to increase life skills (i.e., decision-making, proactivity) among children. Preliminary findings suggest that participating in ACT programs could help custodial, foster and biological parents improve self-efficacy, emotional well-being, higher self-confidence, social competence, lower depressive symptoms, and parenting distress, thereby leading to positive outcomes such as improved mental health and higher resilience.

